# Whole Genome Sequencing Reveals Presence of High-Risk Global Clones of *Klebsiella pneumoniae* Harboring Multiple Antibiotic Resistance Genes in Multiple Plasmids in Mwanza, Tanzania

**DOI:** 10.3390/microorganisms10122396

**Published:** 2022-12-02

**Authors:** Vitus Silago, Stephen E. Mshana

**Affiliations:** Department of Microbiology and Immunology, Weill Bugando School of Medicine, Catholic University of Health and Allied Sciences, Mwanza P. O. Box 1464, Tanzania

**Keywords:** antibiotic resistance genes, biocides, *Klebsiella pneumoniae*, plasmid replicons, quaternary ammonium compound

## Abstract

Background: *Klebsiella pneumoniae* is an important multidrug-resistant (MDR) pathogen, causing both community- and healthcare-associated infections. The resistance is due to the continuous accumulation of multiple antibiotic-resistance-genes (ARGs) through spontaneous genomic mutations and the acquisition of conjugative plasmids. This study presents antibiotics resistance genes, plasmids replicons, and virulence genes of *K. pneumoniae* isolates from clinical specimens in a tertiary hospital, Mwanza, Tanzania. Methods: Whole genome sequencing (WGS) of 34 *K. pneumoniae* was performed, using an Illumina NextSeq 500, followed by in silco analysis. Results: A total of 34 extended-spectrum beta-lactamase-producing *K. pneumoniae*, isolated from blood samples from neonatal units were whole-genome sequenced. Of these, 28 (82.4%) had an identified sequence type (ST), with ST14 (39.3%, n = 11) being frequently identified. Moreover, 18 (52.9%) of the bacteria harbored at least one plasmid, from which a total of 25 plasmid replicons were identified with a predominance of IncFIB(K) 48.0% (n = 12). Out of 34 sequenced *K. pneumoniae*, 32 (94.1%) were harboring acquired antibiotic/biocides-resistance-genes (ARGs) with a predominance of *bla*_CTX-M-15_ (90.6%), followed by *oqx*B (87.5%), *oqx*A (84.4%), *bla*_TEM-1B_ (84.4%) and *sul*2 (84.4%). Interestingly, we observed the ColRNAI plasmid-replicon (n = 1) and *qac*E gene (n = 4) for the first time in this setting. Conclusion: Global high-risk clones of *K. pneumoniae* isolates carry multiple ARGs in multiple plasmid-replicons. Findings from this study warrant genomic-based surveillance to monitor high-risk global clones, epidemic plasmids and ARGs in low- and middle-income countries.

## 1. Introduction

*Klebsiella pneumoniae* is an opportunistic nosocomial pathogen that is responsible for a number of infectious diseases, namely urinary tract infections (UTIs), skin and soft tissue infections (SSTIs), both community- and health-care-associated pneumonia, and blood-stream infections (BSIs) [1,2,3]. Infections by *K. pneumoniae* are reported to be associated with increased health-care costs from treatment failure and prolonged hospitalization, which in turn results in a high risk of mortality [4]. Recently, *K. pneumoniae* has been reported as the commonest member of Enterobacterales, with high rates of resistance towards beta-lactams, aminoglycosides, quinolones, and folate-pathway antagonists antibiotics [5]. *K. pneumoniae* is an important multidrug-resistant (MDR) pathogen, which continuously accumulates for itself multiple antibiotic-resistance-genes (ARGs) through spontaneous genomic mutations and acquisitions of transferable genetic elements, particularly conjugative plasmids [4]. Therefore, *K. pneumoniae* has been identified as the major source and shuttle for antibiotic resistance genes [4].

A report from the Infectious Diseases Society of America listed ESBL-producing *Klebsiella pneumoniae* as one of the six pathogens for which new therapies are urgently needed [6]. Furthermore, the WHO has identified MDR *K. pneumoniae* as a critical-priority pathogen. New antibiotics are urgently needed, because MDR *K. pneumoniae* isolates pose a threat in hospitals among patients requiring devices such as ventilators and blood catheters, and have also been found to cause community infections. Limited treatment-options are available in low- and middle-income countries, and these drugs are too expensive to be afforded by the majority of the population [7,8]. Due to the increasing importance of multi-resistant MDR *K. pneumoniae* in the community and hospital settings, genomic data to track the clones, acquired ARGs, and virulence genes, are of critical importance.

A number of sequence types (STs) of *K. pneumoniae* including ST11, ST14, ST15, ST26, ST37, ST45, ST101, ST147, ST149, ST231, ST258, ST627, and ST977 have been reported, some of which are geographically localized, while others are globally epidemic [9]. *K. pneumoniae* ST11, ST13, ST14, ST15, ST37, ST45 and ST147 are well disseminated globally [10,11,12,13]. On the other hand, replicons belonging to conjugative FIB(K), FII(K), IncHI, IncR, IncQ and Col plasmids are the commonest reservoirs of acquired ARGs among *K. pneumoniae* [9,11,14]. Genes, namely *aac*(3)-IIa and *aac*(6′)-*Ib-cr* for aminoglycosides and quinolones resistance; *aph*(3′′)-Ib, *aph*(6)-Id, and *aph*(3′)-Ia for aminoglycosides resistance; *bla*_OXA-48_, *bla*_NMD_, *bla*_CTX-M-15_, *bla*_OXA-1_ and *bla*_TEM-1B_ for β-lactams resistance; *dfr*A14, and *dfr*A7 for trimethoprim resistance; *sul*1 and *sul*2 for sulfamethoxazole resistance; *qnr*B1 for quinolones resistance; *tet*(A) for tetracycline resistance; and *qac*E for biocides resistance are among the multiple genes harbored in *K. pneumoniae* [5,13]. Moreover, a group of virulence traits: adhesion encoded by *fim*H-1 and *mrk*D genes; iron-sequestering proteins (siderophores) encoded by *fyu*A, *iro*BCDN, *irp*1/2, and *iuc*ABCD genes; complement resistance encoded by *tra*T gene; heavy-metal resistance, e.g., tellurium-ion-resistance protein encoded by *ter*C gene; plasmid-encoded enterotoxin encoded by *sen*B gene; outer-membrane proteins encoded by *Omp*K35/36 genes; and glutamate decarboxylase protein encoded by the *gad* gene, have been reported in *K. pneumoniae* [1,15,16,17,18]. 

The above information justifies the fact that *K. pneumoniae* is the main pathogen that is threatening the effectiveness of the treatment of infectious diseases during this era of global emergence and increasing antibiotic-resistance. Therefore, genomic-based sequencing studies are mandatory in monitoring the epidemic plasmids and resistance genes in *K. pneumoniae,* and here, we present data of acquired ARGs, plasmid-replicons types and virulence genes, related to *Klebsiella pneumoniae* clinical isolates, from a tertiary hospital in Tanzania. 

## 2. Materials and Methods

### 2.1. Laboratory Procedures 

A total of 34 *Klebsiella pneumoniae* isolated between 2012 and 2015 with resistance towards third-generation cephalosporins were retrieved and whole-genome sequenced. The bacteria were isolated from blood samples of neonates who were admitted to neonatal units (neonatal ICU and premature unit) between 2012 and 2015, presenting with signs and symptoms of bloodstream infections. Conventional bacteriological culture, in-house biochemical-identification testing, and the disk-diffusion method using the Kirby–Bauer technique [19] were used for isolation, identification and antibiotics-susceptibility testing of *K. pneumoniae,* respectively. The Clinical and Laboratory Standards Institute (CLSI) guidelines were used for the interpretations of the zone of inhibition [20]. The isolates were archived at −80 °C in the Microbiology Research Laboratory at the Catholic University of Health and Allied Sciences (CUHAS).

For DNA extraction prior to sequencing, *K. pneumoniae* isolates were recovered by plating on plates of Columbia Blood Agar (Becton Dickinson GmbH, D-69126 Heidelberg, Germany) and then incubated aerobically at 35 ± 2 °C, for 20 h.

DNA extraction and purification was carried out using QIAmp® DNA Mini kit (QIAGEN, Hilden, Germany). Briefly, a loopful of 1 µL was used to transfer bacterial colonies into a 1.5 mL safe-lock microcentrifuge tube containing 500 µL nuclease-free ultra-pure molecular water, followed by vortex-mixing to make homogenous suspensions. The suspensions were centrifuged at 13,000 rpm for 10 min, and then the supernatants were discarded. Cell pellets were re-suspended in 180 µL of ATL buffer. Further procedures of DNA extractions and purifications were conducted as instructed by the manufacturer. NanoDrop was used to determine the quantity and quality of the extracted and purified DNA samples. 

### 2.2. Whole Genome Sequencing and In-Silico Analysis 

Whole genome sequencing (WGS) was performed on an Illumina NextSeq 500/550 instrument (Illumina, San Diego, CA, USA,) using an Illumina Nextera XT library with 2 × 150 bp paired-end reads. Sequences data (mean length: 5084 kb) were quality checked before being assembled for multi-locus sequence typing (MLST) and phylogenetic-tree construction using Neighbor Joining tree (NJ-tree) algorithm(Center for Demographic and Population Genetics, University of Texas Health Science Center, Houston 77225)., and analyzed for virulence genes (employing VFDB platform) on SeqSphere^+^ v.8.4.0 (Ridom GmbH, Münster, Germany) pipelines [21]. Genes coding for virulence factors with an identity of ≥95% and an alignment of 100% with the reference sequence were considered present in our sequenced isolates. The detection and typing of plasmid replicons [22] and acquired antibiotics resistance genes (ARGs) were performed on Center for Genomic Epidemiology database (https://www.genomicepidemiology.org/services/; accessed on 9 September 2022).

To confirm the carriage of ARGs in plasmids replicons, the plasmid contigs were extracted from the selected WGS of *K. pneumoniae* representing each cluster, using Platon software(Justus-Liebig-Universität Gießen, Giessen, Germany) [23]. Plasmid-born contigs were then examined for carriage of ARGs using ResFinder 4.1((https://cge.food.dtu.dk/services/ResFinder/; accessed on 24 November 2022) server on Center for Genomic Epidemiology [24,25,26].

### 2.3. Systematic Review 

For the comparison of changes and/or persistence of sequence types (STs), acquired ARGs, virulence factors (VRFs) and plasmid replicons, we systematically reviewed our own studies between 2011 and 2022 in Mwanza, Tanzania. We included studies that established sequence types (STs), acquired ARGs, virulence factors (VRFs), and plasmid replicons among Enterobacterales from humans, animals and the environment both colonizing and causing invasive infections.

## 3. Results

### 3.1. General Overview of Genome-Sequenced Klebsiella pneumoniae 

A total of 34 *Klebsiella pneumoniae* isolated from blood samples from neonatal units were whole-genome sequenced. All isolates were extended spectrum beta-lactamase-producing isolates. These isolates were multidrug resistant (MDR), showing resistance to third- and fourth-generation cephalosporin, gentamicin, tetracycline and trimethoprim-sulfamethoxazole. Regarding ciprofloxacin, only 3 (8.8%) isolates were sensitive to this antibiotic, and all isolates were sensitive to meropenem.

Twenty-eight (82.4%) had an identified sequence-type (ST) with ST14 (39.3%, n = 11) being frequently identified. About 64.3% (n = 18) of the identified STs are known as high-risk (HiR) clones; ST14 (n = 11), ST37 (n = 6) and ST340 (n = 1). Moreover, 18 (52.9%) of the bacteria harbored at least one plasmid from which a total of 25 plasmid replicons were identified, with a predominance of IncFIB(K) 48.0% (n = 12). Out of 34 sequenced *K. pneumoniae*, 32 (94.1%) were harboring genes encoding for antibiotics and/or biocides resistance. Moreover, all sequenced *K. pneumoniae* had acquired genes for virulence factors (Table 1). 

### 3.2. Types and Distributions of Resistance Genes in Sequenced-Klebsiella pneumoniae

The majority of *K. pneumoniae* were harboring *bla*_CTX-M-15_ (90.6%; n = 29), followed by *oqx*B (87.5%; n = 28), *oqx*A (84.4%; n = 27), *bla*_TEM-1B_ (84.4%, n = 27) and *sul*2 (84.4%, n = 27), out of 32 isolates found to harbor genes encoding for antibiotics and/or biocides resistance (Table 2). 

### 3.3. Descriptions of Acquired Virulence Genes among Sequenced K. pneumoniae 

**Figure 1:**
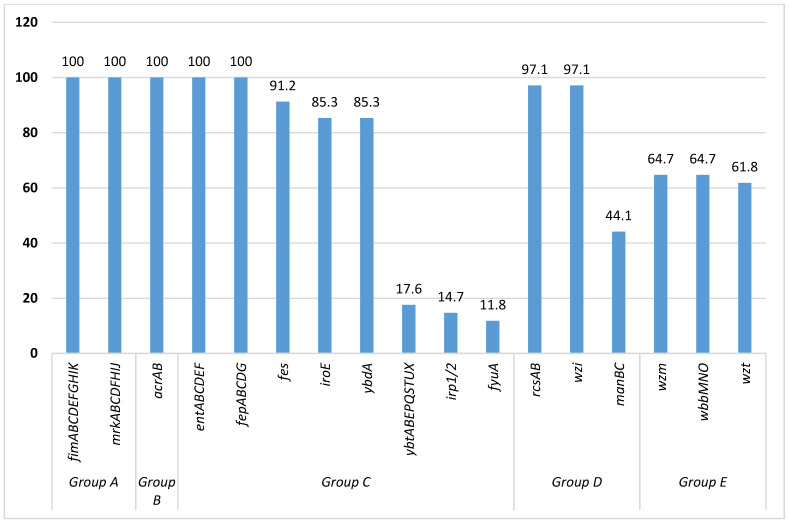
Percentages of virulence genes found in sequenced *Klebsiella pneumoniae*.

We grouped the virulence factors (VFs) into five groups, namely group A (attachment/adhesion factors), group B (plasma/protein-resistant factors), group C (iron-sequestering factors), group D (capsule factors) and group E (lipopolysaccharide O-antigen). All of the sequenced isolates were harboring *fim*ABCDEFGHIK and *mrk*ABCDFHIJ genes, encoding for adhesion; *acr*AB genes encoding for plasma/protein resistance; and *ent*ABCDEF and *fep*ABCDG genes encoding for iron acquisition (100%; n = 34 each). Further, the majority of isolates were harboring *fes* (91.2%; n = 31), *iro*E (85.3%; n = 29), and *ybd*A (85.3%; n = 29) genes, encoding for iron acquisition; *rcs*AB and *wzi* genes (97.1%; n = 33 each) encoding for capsule-formation; and *wzm* (64.7%; n = 22), *wbb*MNO (64.7%; n = 22), and *wzt* (61.8%; n = 21) genes, encoding for lipopolysaccharide O-antigen (Figure 1). 

### 3.4. Clonal Distributions of Sequenced K. pneumoniae

**Figure 2:**
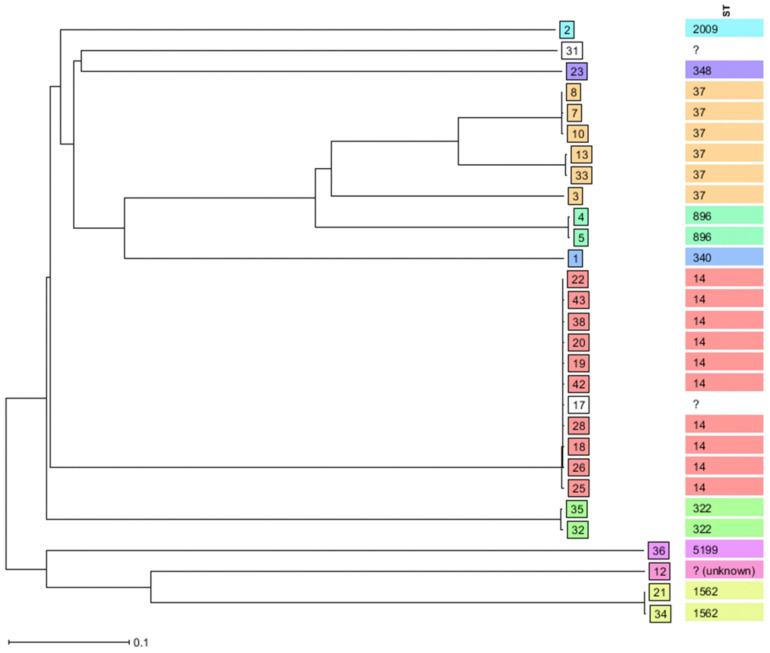
Clonal distribution by Neighbor-Joining tree (NJ tree) algorithm of sequenced *K. pneumoniae* isolated from neonates’ blood samples between 2012 and 2015 by SeqSphere^+^ v.8.4.0 database.

The phylogenetic tree constructed by the NJ tree algorithm showed six MLST clonal-clusters. Those MLST clonal-clusters include: cluster 1, made with *K. pneumoniae* ST14 (n = 10) and unknown ST (n = 1); cluster 2, made with *K. pneumoniae* ST37 (n = 3); cluster 3, made with *K. pneumoniae* ST37 (n = 2); cluster 4, made with *K. pneumoniae* ST322 (n = 2); cluster 5, made with *K. pneumoniae* ST896 (n = 2); and cluster 6, made with *K. pneumoniae* ST1562 (n = 2). Unidentified STs are denoted by question marks (Figure 2).

### 3.5. Sequence Types (STs), Antibiotic Resistance Genes (ARGs), Virulence Genes (VRGs) and Plasmid Replicons Circulating between 2011 and 2022 in Mwanza among Enterobacterales 

In the nine previous studies reviewed, the majority involved humans (n = 6 studies), working with blood samples (n = 4 studies). These studies focused on *E*. *coli* (n = 8 studies) and *K. pneumoniae* complex (n = 7 studies). In characterizing the isolates, conventional PCR and sequencing was used in five studies, while WGS was used in four studies. 

*K. pneumoniae* from Blood (n = 61), pus/wound swabs (n = 19) and urine (n = 12), isolated between 2009 and 2010 using PCR and sequencing showed the predominance of ST14. Other STs detected were ST101, ST48, ST348 and ST147. These isolates were found to carry predominantly *bla*_CTX-M-15_ in conjugative plasmids of IncFII and IncFIA. Other resistance genes detected were *bla*_SHV-11_, *bla*_TEM-10_ and *bla*_TEM-170_. Using WGS, the three *K. pneumoniae* from fish isolated in 2015 were typed as ST37 (n = 2) and ST280. These isolates were found to carry *bla*_CTX-M-15_ and other resistance genes such as *str*A/B, *qnr*S1, *aac*(3)-IIa and *aac*(6′)-Ib-cr in multiple plasmids of replicon types IncFII, IncFIB(K), IncHI1B and IncR. Furthermore, the 38 *K. pneumoniae* from neonates (15 blood and 23 swabs), isolated in 2016, were predominantly typed as ST45 (seven from blood and eleven from swabs), using WGS. Other high-risk clones detected were STs ST348 (n = 3), ST101 and ST14. Multiple acquired ARGs such as *bla*_CTX-M-15_, *bla*_SHV-1_, *bla*_TEM-1B_, *bla*_OXA-1_, *oqx*A, *oqx*B, *qnr*B2, *aac*(6′)Ib-cr, *qnr*S1, *aad*A2 *sul*2 and *dfr*A1 were found in plasmid with replicon types InFIB, IncFII, IncFR, IncHI1B, IncR and IncHI1A. The virulence genes involved in iron-acquisition, such as *irp*1, *irp*2, *fyu*A, etc., were detected in the majority of *K. pneumoniae* isolates. The *bla*_CTX-M_ gene was found to be predominant in *K. pneumoniae* from humans (blood, rectal swabs, urine, stool) and the environment in those isolated between 2018 and 2021.

Regarding the STs, ARG, plasmid replicons and virulence genes from other Enterobacterales in the study setting, a similar picture was observed as that for *K. pneumoniae*. Using WGS, *E. coli* from humans, the environment and animals, global clones such as ST131, ST38, ST617, ST648 and ST10 were observed circulating in humans, animals and the environment. *E.coli* strains harbored multiple acquired ARGs similar to those observed in *K. pneumoniae*. These genes were located in multiple plasmids of replicon types IncI2, Col156, IncFIA, IncFIB, IncFII, IncQ1, and IncY. 

Changes in the predominance of *K. pneumoniae* STs were observed between 2010 and 2016. However, there was a persistence of acquired ARGs and conjugative plasmid circulating among Enterobacterales and other species such as *Acinetobacter* spp. and *Pseudomonas* spp. (Supplementary Material Table S1) [10,11,27,28,29,30,31]. 

### 3.6. Plasmids Harboring Genes Encoding for Antibiotics Resistance 

We observed that extracted plasmid-contigs of the sequenced *K. pneumoniae* were harboring ARGs, conferring resistance to beta-lactams, aminoglycosides, sulfamethoxazole, trimethoprim, tetracyclines, and quinolones, and the biocides, ammonium quaternary compounds (Table 3).

## 4. Discussion

The *Klebsiella pneumoniae* isolated between 2012 and 2015 from blood samples confirmed the existence of global high-risk (HiR)-clones ST14, ST37 and ST340, carrying multidrug-resistance genes in multiple plasmid-replicons in Tanzania, causing invasive infections among neonates. Furthermore, the predominance of the HiR clone ST14, causing invasive infections in neonates was observed in 2009. However, in 2016 other HiR clones, ST 45, ST17, ST20 and ST48 were found to be more predominant in the same units, causing invasive infections and colonizing neonates [11]. 

The insight from our own studies and the findings from the present study show the persistence of *K. pneumoniae* ST14 from 2009 in the neonatal units, as observed by Mshana et *al* 2011 [10] through 2015. Changes in the predominance of *K. pneumoniae* ST in the neonatal units were observed and documented by Marando et *al* in 2018, who observed ST45 and ST35 to be the predominant STs in 2016 [11]. Furthermore, we observed the persistence of acquired ARGs circulating among HiR clones of *K. pneumoniae* and *E. coli*, especially *bla*_CTX-M-15_ and other quinolones and aminoglycosides genes. These acquired ARGs were found in similar conjugative-plasmid replicon types, pointing to the horizontal transfer of these genes among Enterobacterales. The persistence of a clone in the hospital setting can be due to poor infection-prevention practices in the presence of carriers of these clones. These HiR *K. pneumoniae* clones are present beyond the hospital settings, as observed by Moremi et *al*. in 2015, who found that the HiR clone ST37 was found to colonize the gut of tilapia fish in the same region [27]. 

As observed in this study and by Marando et *al* [11] and another study in Germany, HiR *K. pneumoniae* clones ST14, ST15, ST17, ST20, ST37, ST48, ST147 and ST307 are multidrug resistant (MDR) and are predominantly colonizing and causing sepsis in both hospital and community settings [28]. These clones of the *K. pneumoniae* complex have a global distribution and a potential association with outbreaks of health-care-associated infections [28,29]. 

*K. pneumoniae* ST14 and ST37 have been found to carry high levels of colistin-resistance and carbapenem-resistance genes such as *bla*_NDM_ and *bla*_OXA-48_ [30,31]; however, these genes were not observed in the current study or in previous studies that used WGS from the same setting [10,11,27]. This fact therefore underscores the need for the continuous genomic surveillance of MDR pathogens in the community and in hospital settings, for the early detection of the changes in clones and the acquisition of other acquired ARGs. 

Eighteen out of 34 sequenced *K. pneumoniae* in the current study were harboring mobile genetic elements, notably plasmids of various replicon types including ColRNAI, which was observed in the clinical isolates of *K. pneumoniae* at our setting for the first time. Similar common replicon-types were observed among Enterobacterales in the study setting, with a predominance of conjugative IncFII and IncFIB plasmids (Supplementary Material Table S1). The ColRNAI was detected in one *K. pneumoniae* ST340. *K. pneumoniae* ST340 with ColRNAI plasmid was also harboring other plasmids, including IncFII(K), IncFIB(K) and IncR, of which altogether were carrying acquired ARGs, namely *aac*(6′)-Ib-cr, *aad*A2, *aph*(3′)-Ia, *bla*_CTX-M-15_, *bla*_OXA-1_, *Oqx*A, *Oqx*B, *qac*E, *sul*1 and *tet*(A). A recent study in Japan reported that *K. pneumoniae* ST258, harboring ColRNAI plasmid [32] reserves multiple ARGs, as observed in the current study.

We observed some isolates harboring more than one different plasmid-replicon, with one isolate carrying four plasmids, namely ColRNAI, IncR, IncFII(K), and IncFIB(K). As previously described [11,33] and observed in our previous studies (Supplementary Material Table S1), the commonest replicon types are IncY and IncF i.e., IncFIA, IncFIB and IncFII. The IncFIB and IncFII(K) replicons are commonly reported in clinical isolates of *K. pneumoniae* and *E.coli*, and are highly associated with the dissemination of acquired ARGs [34]. On the other hand, we identified two *K. pneumoniae* with IncHI2/2A plasmid-replicons, despite the fact that IncHI2/2A replicons have been identified as predominant reservoirs of acquired ARGs in multidrug-resistant *Salmonella* spp. [32,35]. Our findings are also supported by a recent study which observed IncHI1B as the commonest plasmid among *K. pneumoniae* isolated from blood samples [36]. Rare plasmids of replicon types IncQ1 and IncR were also identified in the current study and in other previous studies in the same setting (Supplementary Material Table S1). IncQ1 and IncR plasmids have been found to carry multiple resistance-genes, such as *bla*_NDM-1_, *bla*_KPC-2_, *bla*_DHA-1_, *bla*_VIM-1_, *qnr*S1, or *arm*A [37,38]. 

Similarly to previous studies from the same setting [10,11], *bla*_CTX-M-15_ and *bla*_TEM-1B_ conferring resistance towards β-lactams, *sul*2 conferring resistance to sulfamethoxazole; *aph*(3”)-Ib, and *aph*(6)-Id conferring resistance to aminoglycosides were the commonest acquired ARG genes detected in the sequenced *K. pneumoniae*. However, this is the first study to report *bla*_LEN10_ and *qac*E genes conferring resistance towards β-lactam antibiotics and quaternary ammonium compound, respectively, in *K. pneumoniae*, in this setting. Our findings support observations by Damiano and colleagues, who reported phenotypic resistance of Enterobacterales towards antiseptics/biocides, in this setting [39]. The *bla*_LEN10_ has been previously reported among the environmental isolates of *Klebsiella* spp. in Nigeria [40] and the USA [41]. The presence of *qac*E genes was also reported previously in *K. pneumoniae* from one study in Iran [42] and another study in the UK [43]. The *bla*_LEN10_ gene was identified among two *K. pneumoniae* of the ST1562 type; however, the only IncR plasmid which was present in these isolates did not carry this gene. The *bla*_LEN_ has been identified as class A chromosomal Beta-Lactamase gene in *Klebsiella pneumoniae* previously [44]. On the other hand, the four *qac*E genes were identified among three *K. pneumoniae* of STs 340, 5199 and 14 and one *K. pneumoniae* with an unknown ST. 

The acquired virulence-genes in the current study were categorized into five groups, namely adhesins (group A), plasma/protein-resistance factors (group B), iron-acquisition/sequestering system (group C), capsule-formation factors (group D), and lipopolysaccharide O-antigen (group E) [45,46,47]. However, the majority of our sequenced isolates were harboring *fim*ABCDEFGHIK and *mrk*ABCDFHIJ genes, encoding for adhesion/attachment, an essential first step in developing infectious disease; *ent*ABCDEF, *fep*ABCDG, *fes*, *iro*E, and *ybd*A genes, encoding for iron acquisition from host tissues, which ensures the availability of essential micronutrients for bacterial survival as well as the destruction of host tissues; *acr*AB genes that encode for plasma/protein resistance to facilitate evading the host immune-response; *rcs*AB and *wzi* genes, encoding for capsule formation and facilitating the evading of the host immune-response through anti-phagocytosis; and *wzm*, *wzt*, and *wbb*MNO genes, responsible for hypervirulence [45,46,47]. Other virulence-encoding genes such as *irp*1/2 and *fyu*A were reported in the same setting by Marando and colleagues; these virulence genes are responsible for iron acquisition from the environment [11], and might indicate that these *K. pneumoniae* might be from the community in origin [48]. Moreover, *acr*AB, *man*BC, *wzi*, *wzt*, and *wzm* genes were reported for the first time among clinical isolates of *K. pneumoniae* in this setting.

The neighbor-joining tree (NJ tree) algorithm grouped sequenced *K. pneumoniae* into six MLST clonal-clusters. The large MLST clonal-cluster is made with 11 *K. pneumoniae,* of which 10 belong to ST14 and 1 is of unidentified ST. Other MLST clonal-clusters are made of three *K. pneumoniae* (n = 1; ST37); and two *K. pneumoniae* (n = 2 each; ST37, ST322, ST896 and ST1562). These findings indicate the possible cross-transmission of *K. pneumoniae*, particularly ST14, ST37, ST322, ST896 and ST1562, causing neonatal sepsis in neonatal units in this setting. Similar findings were reported previously, from the same setting [49]. Therefore, the stringent implementation of IPC measures is mandatory for minimizing the spread of HiR clones, i.e., *K. pneumoniae* ST14 and ST37, causing neonatal sepsis in this setting. 

## 5. Conclusions

*K. pneumoniae* ST14 harboring hypervirulent genes (*wzm*, *wzt*, and *wbb*MNO) and multiple acquired-ARGs is an important and successful disseminated sequence-type among clinical isolates, causing bloodstream infections in our setting between 2009 and 2015, with changes in 2016, whereby the ST45 was predominant. The FIB(K) and FII(K) plasmid replicons carrying *bla*_CTX-M-15_, *sul*2 and *aph*(3”)-Ib and *aph*(6)-Id ARGs were predominantly identified in the high-risk global clones of *K. pneumoniae* from this setting. The less-common plasmid-replicon ColRNAI, and *qac*E and *bla*_LEN10_ genes were also reported for the first time among clinical isolates of *K. pneumoniae* in this setting. The majority of isolates are harboring genes encoding for the iron-sequestering system that can indicate the community spread of MDR pathogens. The predominance and the persistence of the *bla*_CTX-M-15_ gene and other acquired-ARGs among Enterobacterales in our setting might be due to circulating conjugative-plasmids and the spread of HiR-clones of *K. pneumoniae* in the hospital setting, due to poor IPC-practices. These findings warrant sustained improved IPC-practices and genomic-based sequencing surveillance to monitor *K. pneumoniae* HiR clones, epidemic plasmids, and virulence and acquired antibiotic-resistance genes among MDR pathogens causing invasive infections in developing countries.

## Figures and Tables

**Table 1:** General overview of genome-sequenced *Klebsiella pneumoniae*.

Characteristics		Frequency (n)	Percentage (%)
Sequence types (STs; N = 34)	Determined STs	28	82.4
	Undetermined STs	6	17.6
Types of STs identified (N = 28)	ST14	11	39.3
	ST37	6	21.4
	ST1562	2	7.1
	ST322	2	7.1
	ST896	2	7.1
	Other STs (ST2009, ST45, ST5199, ST384, ST340)	5	17.9
Bacteria harboring typeable plasmid-replicons (N = 34)	Yes	18	52.9
	No	16	47.1
Amount of typeable plasmid-replicons in each bacteria (N = 18)	1	14	77.8
	2	2	11.1
	3	1	5.6
	4	1	5.6
Types of plasmid replicons (N = 25)	IncFIB(K)	12	48.0
	IncFII(K)	4	16.0
	IncQ1	3	12.0
	IncR	3	12.0
	Others [(IncHI2/2A, n=2) and (ColRNAI, n = 1)]	3	12.0
Antibiotics/biocides-resistance genes	Yes	32	94.1
	No	2	5.9
Acquired virulence genes	Yes	34	100
	No	0	0

**Table 2:** Types and distributions of acquired antibiotic resistance genes (ARGs) in sequenced *Klebsiella pneumoniae*.

Types of Resistance Genes		Frequency (n)	Percentages (%)
Β-lactam resistance	*bla*_CTX-M-15_	29	90.6
	*bla*_TEM-1B_	27	84.4
	*bla*_SHV-100_	10	31.3
	*bla*_SCO-1_	10	31.3
	*bla*_OXA-1_	4	12.5
	*bla*_LEN10_	2	6.3
	*bla*_SHV-148_	1	3.1
Aminoglycosides resistance	*aph*(3′)-Ib	16	50
	*aph*(6)-Id	16	50
	*aph*(3′)-III	1	3.1
	*aph*(3′)-Ia	2	6.3
	*aac*(3)-IIa	15	46.9
	*aac*(3)-IId	13	46.9
	*aac*(6′)-Ib-cr	4	12.4
	*strA*	16	50
	*strB*	16	50
	*aadA1*	2	6.3
	*aadA2*	3	9.4
Trimethoprim resistance	*dfr*A1	1	3.1
	*dfr*A7	1	3.1
	*dfr*A12	2	6.3
	*dfr*A14	4	12.5
	*dfr*A30	13	46.9
	*dfr*G	2	6.3
Sulfamethoxazole resistance	*sul*2	27	84.4
	*sul*1	3	9.4
Quinolones resistance	*Oqx*A	27	84.4
	*Oqx*B	28	87.5
	*qnr*B1	2	6.3
	*qnr*B66	1	3.1
	*aac*(6′)-Ib-cr	2	6.3
Tetracycline resistance	*tet*(A)	4	12.5
	*tet*(M)	2	6.3
	*tet*(L)	1	3.1
Aminoglycosides/quinolones resistance	*aac*(3)-IIa	14	43.8
	*aac*(6′)-Ib-cr	4	12.5
Aminocyclitol/aminoglycosides resistance	*aad*A1	1	3.1
	*aad*A2	1	3.1
Biocides/antibiotics resistance	*Oqx*A	5	15.6
	*Oqx*B	4	12.5
Biocides resistance	*qac*E	4	12.5

**Table 3:** Plasmids harboring genes encoding for antibiotics resistance.

Isolate No.	ST	Plasmids	Antibiotic Classes and Respective Genes Encoding for Their Resistance, Harbored in Plasmids						
			Beta-Lactams	Aminoglycosides	Sulfamethoxazole	Trimethoprim	Tetracyclines	Quinolones	Biocides
ESBL 21	45	IncFIB IncFII	*bla*_CTX-M-15_ *bla*_TEM-1B_ *bla*_OXA-1_	*aac*(6′)-Ib-cr *aph*(3″)-Ib *aac*(3)-IIa *aph*(6)-Id	*sul*2	*dfr*A14	*tet*(A)	*aac*(6′)-Ib-cr *qnr*B1	
ESBL 88	1562	IncR	*bla*_CTX-M-15 _ *bla*_TEM-1B_	*aac*(3)-IIa	*sul*2				
ESBL 219	322	IncFIB IncFII IncR	*bla*_CTX-M-15 _ *bla*_TEM-1B_	*aac*(3)-IId					
ESBL 428	1155	IncFII IncQ1 IncR	*bla*_CTX-M-15 _ *bla*_TEM-1B_	*aph*(3″)-Ib *aac*(3)-IId	*sul*1	*dfr*A7 *dfr*A30			*qac*E
ESBL 703	14	IncFIB IncFII	*bla*_CTX-M-15 _ *bla*_TEM-1B _ *bla*_SCO-1_	*aac*(3)-IIa *aph*(6)-Id *aph*(3″)-Ib *aac*(3)-IId	*sul*2				
ESBL 1270	340	IncF ColRNAI IncR	*bla*_CTX-M-15 _ *bla*_OXA-1_	*aac*(6″)-Ib-cr *aph*(3″)-Ia *aad*A2	*sul*1	*dfr*A12	*tet*(A)		*qac*E
ESBL 1358	37	IncFII IncR	*bla*_CTX-M-15 _ *bla*_TEM-1B_		*sul*2				

## Data Availability

The data presented in this study are available on request from the corresponding author. The sequence data have been deposited in the database with accession numbers ERR829918-ERR829962 (https://www.ebi.ac.uk/ena/browser/view/ERR829918-62, accessed on 22 November 2022).

## Supplementary Materials

The following supporting information can be downloaded at: https://www.mdpi.com/article/10.3390/microorganisms10122396/s1, Supplementary Table S1: Sequence types (STs), antibiotic resistance genes (ARGs), virulence factors (VRFs) and plasmid replicons circulating between 2011 and 2022 in Mwanza.
