# Supplementary material for: Whole Genome Sequencing Reveals Presence of High-Risk Global Clones of Klebsiella pneumoniae Harboring Multiple Antibiotic Resistance Genes in Multiple Plasmids in Mwanza, Tanzania
Source: Microorganisms. 2022 Dec 2;10(12):2396. doi: 10.3390/microorganisms10122396 (PMC9785957; doi:10.3390/microorganisms10122396)
Supplement: Supplementary file 1 [file microorganisms-10-02396-s001.zip › microorganisms-2021208-supplementary.pdf]

Supplementary Material Table S1: Sequence types (STs), antibiotic resistance genes (ARGs), virulence factors (VRFs) and plasmid replicons circulating between 2011 and 2022 in Mwanza.

Author and year	Population	Samples	Year of collection	Method	Bacteria species	ST	Resistance genes	Virulence genes	Plasmid replicons	Reference
**Mshana et al., 2013**	Humans	Blood<br>Urine<br>Pus<br>wound swab	April 2009 – March 2010	PCR<br>PFGE	*K. pneumoniae* complex	ST14<br>ST101<br>ST48<br>ST348<br>ST147	*bla*$_{\text{CTX-M-15}}$<br>*bla*$_{\text{TEM-1}}$<br>*bla*$_{\text{TEM-10}}$<br>*bla*$_{\text{SHV-11}}$<br>*bla*$_{\text{TEM-170}}$	NA	IncFII<br>IncFIA	[10]
**Seni et al., 2016**	Animals	Rectal swab<br>cloaca swab	August – September 2014	WGS	*E. coli*	ST10<br>ST44<br>ST617<br>ST38<br>ST131<br>ST410<br>ST1642<br>ST256<br>ST2852<br>ST1421<br>ST1598<br>ST4977<br>ST746<br>ST1303<br>ST5455	*bla*$_{\text{CTX-M-15}}$<br>*bla*$_{\text{TEM-1B}}$<br>*bla*$_{\text{OXA-1}}$<br>*str*A<br>*str*B<br>*aac*(3)-IId<br>*aac*(3)-IIa<br>*aad*A5<br>*aad*A1<br>*aad*A2<br>*aac*A4<br>*aac*(6′)-Ib-cr<br>*qnr*S1<br>*sul*1<br>*sul*2<br>*dfr*A14<br>*dfr*A17<br>*tet*(A)<br>*tet*(B)	NA	IncFIA<br>IncFIB<br>IncFII<br>IncY	[25]
**Mshana et al., 2016**	Humans	Stool	June – September 2014	WGS	*E. coli*	ST648<br>ST4450<br>ST2852<br>ST617<br>ST38<br>ST131<br>ST205<br>ST44	*bla*$_{\text{CTX-M-15}}$<br>*bla*$_{\text{OXA-1}}$<br>*bla*$_{\text{TEM-1B}}$<br>*aad*A4<br>*aad*A4<br>*aac*(6′)-Ib-cr<br>*aac*(3)-IIa<br>*qnr*S1<br>*str*A<br>*str*B	*gad*<br>*iss*<br>*sat*<br>*iha*<br>*ipf*A<br>*nfa*E<br>*sen*B<br>*cnf*1<br>*ire*A	IncFIA<br>IncFIB<br>IncFII<br>IncY<br>IncQ1	[26]
**Moremi et al., 2016**	Tilapia fish<br>Community environment	Wastewater<br>Fish gut	July - September 2015	WGS	*E. coli*	ST38<br>ST5173<br>ST2852<br>ST1049<br>ST1421<br>ST131<br>ST10<br>ST394<br>ST1177<br>ST58<br>ST167<br>ST48	*bla*$_{\text{CTX-M-15}}$<br>*bla*$_{\text{TEM-1B}}$<br>*bla*$_{\text{ACT-15}}$<br>*bla*$_{\text{OXA-1}}$<br>*bla*$_{\text{MIR-3}}$<br>*bla*$_{\text{CMY-37}}$<br>*bla*$_{\text{SHV-11}}$<br>*bla*$_{\text{CMY-49}}$<br>*bla*$_{\text{CTX-M-55}}$<br>*dfr*A14<br>*dfr*A18<br>*dfr*A30	NA	IncFII<br>IncFIB<br>IncR<br>IncI1<br>IncY<br>IncHI1B<br>IncP<br>IncFIA<br>IncQ1	[27]

					E. cloacae<br><br><br>*K. pneumoniae complex*	ST91<br>ST422<br>ST500<br>ST37<br>ST280	*dfr*A7<br>*dfr*A17<br>*dfr*A1<br>*dfr*A5<br>*sul*1<br>*sul*2<br>*aad*A1<br>*aac*(6′)-Ib-cr<br>*aac*(3)-IIa<br>*aad*A2<br>*str*B<br>*str*A<br>*acc*(3)-IId<br>*aad*A5<br>*qnr*B1<br>*qnr*B29<br>*qnr*S1<br>*oqx*A<br>*oqx*B<br>*qnr*B48<br>*tet*(A)<br>*tet*(D)			
**Moremi et al., 2017**	Humans	Stool	April – July 2015	PCR and Sequencing	*E. coli*	ST10<br>ST1585<br>ST167<br>ST617<br>ST34<br>ST48<br>ST1284<br>ST398<br>ST1421<br>ST5173<br>ST648<br>ST2852<br>ST156<br>ST131<br>ST38<br>ST448<br>ST405<br>ST1177	*bla*$_{\text{CTX-M-15}}$<br>*bla*$_{\text{CTX-M-9}}$<br>*bla*$_{\text{CTX-M-55}}$<br>*bla*$_{\text{TEM-1}}$<br>*bla*$_{\text{SHV-1}}$<br>*bla*$_{\text{SHV-11}}$	NA	NA	[28]
**Marando et al., 2018**	Humans	Blood umbilical-rectal swab	July – December 2016	WGS	*K. pneumoniae* complex<br><br><br><br><br><br><br><br><br><br><br>*E. coli*	ST101<br>ST348<br>ST35<br>ST45<br>ST48<br>ST14<br>ST17<br>ST20<br>ST2268<br>ST711<br>ST873<br>ST131<br>ST405	*bla*$_{\text{CTX-M-15}}$<br>*bla*$_{\text{SHV-1}}$<br>*bla*$_{\text{TEM-1B}}$<br>*bla*$_{\text{SHV-11}}$<br>*bla*$_{\text{SHV-33}}$<br>*bla*$_{\text{SCO-1}}$<br>*bla*$_{\text{SHV-28}}$<br>*bla*$_{\text{OXA-1}}$<br>*bla*$_{\text{SHV-83}}$<br>*bla*$_{\text{SHV-27}}$<br>*bla*$_{\text{ADC-25}}$<br>*bla*$_{\text{OXA-69}}$<br>*bla*$_{\text{NDM-1}}$	*mrk*A<br>*mrk*B<br>*mrk*C<br>*mrk*D<br>*mrk*F<br>*mrk*H<br>*mrk*I<br>*mrk*J<br>*fyu*A<br>*irp*1<br>*irp*2<br>*ybt*A<br>*ybt*E	IncFIA<br>IncFIB<br>IncFII<br>IncR<br>IncHI1B<br>IncFR<br>IncHI1A<br>IncHI2A<br>IncHI2<br>IncI2<br>IncI1<br>Col156<br>IncQ1	[11]

						ST617<br>ST648	*bla*$_{OXA-58}$<br>*bla*$_{OXA-69}$	*ybt*P<br>*ybt*Q<br>*ybt*S<br>*ybt*T<br>*ybt*U<br>*ybt*X<br>*all*B<br>*gcl*<br>*ybb*W<br>*ylb*E<br>*iro*B<br>*iro*C<br>*iro*D<br>*iro*N<br>*iuc*A<br>*iuc*B<br>*iuc*C<br>*iuc*D<br>*iut*A<br>*kfu*A<br>*kfu*B<br>*kfu*C<br>*kvg*A<br>*kvg*S	IncY<br>Col(BS512)	
					A. baumannii	ST405<br>ST1470	*bla*$_{CARB-8}$<br>*bla*$_{ACT-7}$			
					E. cloacae	ST93<br>ST116				
**Silago et al., 2021**	Humans<br>Hospital environment	Blood<br>rectal swab<br>patient bed/cot swab	Dec 2018-July 2019	PCR	*E. coli*<br>*K. pneumoniae* complex	NA	*bla*$_{CTX-M}$<br>*bla*$_{TEM}$<br>*bla*$_{SHV}$	NA	NA	[29]
**Silago et al., 2022**	Humans	Blood<br>Urine<br>rectal swab<br>bed swab	Dec 2018-July 2019	PCR	*E. coli*<br>*K. pneumoniae* complex<br>*Acinetobacter* spp<br>*P. aeruginosa*	NA	*bla*$_{IMP}$<br>*bla*$_{KPC}$<br>*bla*$_{OXA-48}$	NA	NA	[30]
**Said et al., 2022**	Humans	Stool/rectal swab	July – September 2021	PCR	*E. coli*<br>*K. pneumoniae* complex	NA	*bla*$_{CTX-M}$<br>*bla*$_{TEM}$<br>*bla*$_{SHV}$	NA	NA	[31]
